# Dieter Brockmann, Michael Kühl: Mit Erfolg promovieren in den Life Sciences

**DOI:** 10.3205/zma001106

**Published:** 2017-08-15

**Authors:** Michael Gommel

**Affiliations:** 1 Institut für systemische Medizin- und Organisationsethik, Berlin, Germany

## Recension

At first glance the book’s title and the text on its back cover lead one to believe that the book is primarily written for doctoral students or those interested in pursuing doctoral studies. Which doctoral candidate doesn’t want to graduate quickly and successfully? The subtitle printed on the third page, however, indicates that the intended audience is much larger: doctoral students, advisors and universities. Even the table of contents quickly reveals that this book covers more than just the ins and outs of completing a doctoral program.

The first chapter could be described as an encyclopedia entry with statistics and the history of graduate study: definitions are explained, statistics and data are given regarding graduation and its importance over the centuries. In the second chapter, regulatory frameworks, types of institutions, admissions and study procedures are described; all in all, rather briefly if one considers the significance of the formal requirements.

The third chapter purports to discuss the development of modern graduate study but instead the authors focus on describing structured graduate study programs in detail, how they are organized and, above all, their advantages. The authors go on to emphasize a preference for such programs in comparison to individualized doctoral studies. This preference is most probably due to Michael Kühl’s and Dieter Brockmann’s many years of positive experience as head and managing director of an international graduate school.

In the fourth chapter the authors focus on optimal conditions for doctoral study addressing academic, practical and personal factors. The section on academic factors covers the reputation of the academic advisor at length, including explanations of the Hirsch index, impact factor and other supposed “quality criteria.” Whether the reputation of the main academic advisor really does carry great importance in the decision to pursue graduate study in a specific field would be an interesting research question. I find the authors’ claim to be reckless that the better the reputation of the adviser, the easier a subsequent academic career will be if accompanied by great motivation and excellent grades. At least the authors admit that an advisor’s professional reputation does not allow conclusions to be drawn about his or her abilities to advise doctoral students.

The fifth and sixth chapters focus on the practical aspects of graduate study as a project that must be managed with a limited amount of resources. The fifth chapter describes the important aspects dealing with the practical realization of doctoral study: planning, milestones, control mechanisms, publication in journals and writing the dissertation. The seventh chapter lists possible options for after graduation. The eighth chapter provides a brief summary of several laws and regulations that could be relevant for doctoral students in the life sciences (genetic engineering, animal rights, etc.).

In the final chapter the authors cover in depth different aspects of good academic practice and academic misconduct. Topics include keeping lab records and logs, saving and keeping data, rights of use, issues surrounding authorship and the ombudsman. Since there has been little written for doctoral students regarding this aspect, I would like to highlight this chapter, despite its minor deficits. I find it unfortunate that the authors rely on the American definition of misconduct, which covers only data fabrication, manipulation and plagiarism. The definitions of misconduct contained in the formal rules and regulations of German universities and research institutes are much more extensive. Furthermore, graduate students come into contact much more frequently with other forms of misconduct than the three above, namely the so-called honorary authorship for a person who has made no actual contribution to a piece of writing. A positive definition of authorship is also missing, such as the one recommended by the International Committee of Medical Journal Editors. It would be important to add that guidelines, rules and recommendations concerning good academic practice do exist at all German universities and research institutes and are binding for all members. A significant error is present in the statement about the commencement of the ten-year duty to preserve data: this is (fully plausibly) the time point of publication, not data collection or generation, as is correctly pointed out in the Ulm University rules on good academic practice.

This book is ambitious in its aim to provide a comprehensive look at doctoral studies in the life sciences and its focus on all parties involved. I find it helpful for all who are thinking about pursuing graduate studies in the life sciences because it offers an overview of many aspects and topics that can be relevant over the course of graduate study and presents a great deal of factual information. A dozen or so checklists and tables help to identify useful questions regarding preparing and carrying out a doctoral project. The guideline is suitable for reading by all who wish to obtain a general overview of doctoral study. For those involved in the coordination of the graduate study programs the book provides important tips about what should be given attention during planning and monitoring. Since the entire book is limited to only 150 pages and covers so much material, it can’t be expected to cover all of the importance aspects in great detail.

An English translation of this book would be very helpful for graduate students – even those not in the life sciences – who do not speak German or who have little knowledge of the German research scene.

## Competing interests

The author declares that he has no competing interests. 

